# Distributed weighted least-squares estimation with fast convergence for large-scale systems^☆^

**DOI:** 10.1016/j.automatica.2014.10.077

**Published:** 2015-01

**Authors:** Damián Edgardo Marelli, Minyue Fu

**Affiliations:** a School of Electrical Engineering and Computer Science, University of Newcastle, University Drive, Callaghan, NSW 2308, Australia; b Department of Control Science and Engineering and State Key Laboratory of Industrial Control Technology, Zhejiang University, 388 Yuhangtang Road Hangzhou, Zhejiang Province, 310058, PR China; c Acoustics Research Institute, Austrian Academy of Sciences, Austria

**Keywords:** Distributed estimation, Distributed state estimation, Large scale optimization, Sensor network, Networked control

## Abstract

In this paper we study a distributed weighted least-squares estimation problem for a large-scale system consisting of a network of interconnected sub-systems. Each sub-system is concerned with a subset of the unknown parameters and has a measurement linear in the unknown parameters with additive noise. The distributed estimation task is for each sub-system to compute the globally optimal estimate of its own parameters using its own measurement and information shared with the network through neighborhood communication. We first provide a fully distributed iterative algorithm to asymptotically compute the global optimal estimate. The convergence rate of the algorithm will be maximized using a scaling parameter and a preconditioning method. This algorithm works for a general network. For a network without loops, we also provide a different iterative algorithm to compute the global optimal estimate which converges in a finite number of steps. We include numerical experiments to illustrate the performances of the proposed methods.

## 1 Introduction

A sensor network is a web of a large number of intelligent sensing and computing nodes connected via a communication network (Dargie & Poellabauer, 2010). The emergence of sensor networks calls for the development of distributed algorithms for a number of tasks to replace the traditional centralized methods. In particular, the development of distributed algorithms for parameter estimation has recently attracted a great deal of attention (Fang and Li, 2008, Kar et al., 2012, Li and AlRegib, 2007, Li and AlRegib, 2009, Lopes and Sayed, 2008, Ribeiro and Giannakis, 2006a, Ribeiro and Giannakis, 2006b, Ribeiro et al., 2010, Xiao et al., 2006). They find applications in environmental and weather monitoring, industrial process monitoring and control, surveillance, state estimation for smart grid, etc.

Existing distributed estimation methods can be classified in several ways. The first classification of the methods is done according to whether a coordinating node or fusion center (FC) is present. When an FC is present, each node communicates with the FC either directly (via a star topology) or indirectly (via a mesh topology), i.e., there is a directed communication path from either node to the FC. The estimation is carried out at the FC, after some possible pre-processing at each node before transmission. The methods proposed in Fang and Li (2008), Li and AlRegib, 2007, Li and AlRegib, 2009, Ribeiro and Giannakis, 2006a, Ribeiro and Giannakis, 2006b are of this type. On the other hand, when no FC is present, estimation is done by executing a cooperative algorithm among all the nodes of the network. The network is connected in some way and each node communicates with its neighboring nodes only. Representative methods of this type include Conejo, de la Torre, and Canas (2007), Gómez-Expósito, Villa Jaén, Gómez-Quiles, Rousseaux, and Van Cutsem (2011), Kar et al. (2012) and Lopes and Sayed (2008). A second classification is done by whether the estimation method is static or dynamic. In static estimation, a set of parameters is estimated using the measurements of all nodes, which collectively form a snapshot of the system at a fixed time. Examples of this type include Fang and Li (2008), Kar et al. (2012), Li and AlRegib, 2007, Li and AlRegib, 2009, Lopes and Sayed (2008) and Ribeiro and Giannakis, 2006a, Ribeiro and Giannakis, 2006b. On the other hand, in dynamic estimation all the nodes track the evolution of a set of parameters for which a dynamic model is available, as in the Refs. Carli, Chiuso, Schenato, and Zampieri (2008), Hlinka, Sluciak, Hlawatsch, Djuric, and Rupp (2012), Khan and Moura (2008) and Ribeiro et al. (2010). Some “hybrid” methods exist, which permit tracking a time-varying sequence of parameters, without a dynamic model, by updating a static estimate at each time (Lopes & Sayed, 2008). A final classification is done by whether a distributed estimation method is a small-scale one or a large-scale one. In a small-scale method, all nodes estimate a common set of parameters (Fang and Li, 2008, Kar et al., 2012, Li and AlRegib, 2007, Li and AlRegib, 2009, Lopes and Sayed, 2008, Ribeiro and Giannakis, 2006a, Ribeiro and Giannakis, 2006b). But in a large-scale method, each node only reconstructs a subset of the parameters, i.e., the collective knowledge of the parameters is distributed among all the nodes (Conejo et al., 2007, Gómez-Expósito et al., 2011, Huang et al., 2012, Khan and Moura, 2008). Large-scale estimation is in general more challenging than its small-scale counterpart.

In this paper we study distributed static estimation for a large-scale system consisting of a network of interconnected sub-systems. Each sub-system is concerned with a subset of the unknown parameters and has measurements, linear in the unknown parameters, corrupted by additive noise. The distributed estimation task is for each sub-system to estimate its local state using its own measurement and information shared with the network through neighborhood communication. We use the weighted least squares (WLS) criterion for optimal estimation. The goal is that the composite estimate of the whole system, consisting of all local estimates, will become globally optimal in the sense that it is the same as the optimal estimate obtained using all the measurements and a centralized estimation method.

This problem is motivated by many applications involving a large-scale spatially distributed system. For example, the state estimation problem for a large power network is concerned with estimating the voltages and phases of the power supply at each sub-system, consisting of a number of buses or a substation, using measurements (provided by, for example, a phasor measurement unit (PMU) or a supervisory control and data acquisition (SCADA) system) (Conejo et al., 2007, Jiang et al., 2008). Interactions of sub-systems are reflected by the fact that local measurements available at each sub-system typically involve neighboring sub-systems. For example, a current measurement at a conjunction depends on the voltage difference of two neighboring buses. In a smart grid setting, each sub-system is only interested in the local state, i.e., its own voltages and phases, using local measurements and information acquired from neighboring sub-systems via neighborhood communication (Tai, Marelli, Rohr, & Fu, 2013). For a large power network, it is both impractical and unnecessary for every sub-system to estimate the whole state of the system, thus distributed estimation methods for local state estimation are naturally called for. The second example is the localization problem for a wireless sensor network, involving estimating the locations of all sensors using relative measurements (e.g., relative distances or relative positions) between sensors (Diao et al., 2013, Khan et al., 2009). For a small sensor network with a few sensing nodes, it is possible to aggregate all the measurements at an FC to compute a global estimate of all sensor locations. Such an algorithm is not scalable, and would require massive computing resources when the network becomes large. It is also unnecessary for each sensor to localize other nodes. A distributed method is preferred, where each node aims to estimate its own location using local measurements and neighborhood communication. The third example is a traffic network for a city or a highway system, where each node or sub-system wants to estimate its local state (e.g., traffic flow rates, delays, etc.). Due to the spatial correlations of the traffic flows in different sub-systems, neighboring traffic information is certainly useful in predicting the traffic conditions at each sub-system. Again, distributed estimation methods are preferred over centralized methods. Many other examples in sensor networks can be found in, e.g., Ribeiro et al. (2010), Kar et al. (2012) and Yang et al. (2010).

We first provide a fully distributed iterative algorithm for each node to compute its own local estimate. This algorithm works for a general connected network. Contrary to the method proposed in Conejo et al. (2007), our algorithm guarantees that the composite estimate will converge asymptotically to the global WLS estimate. We then focus on the convergence rate of the algorithm. Since our method is based on Richardson’s method for solving systems of linear equations (Bertsekas & Tsitsiklis, 1997), its convergence rate depends on certain scaling parameter and a preconditioning matrix. Choosing the optimum scaling parameter requires the knowledge of the largest and the smallest eigenvalues of certain positive definite matrix (the estimation error covariance). A distributed algorithm for estimating these values can be obtained using the power method (Bertsekas & Tsitsiklis, 1997). However, to prevent numerical instability, this approach requires periodically executing a normalization step, which needs to be carried out in a distributed manner. In Yang et al. (2010), this is done using average consensus. A drawback of this approach is that consensus itself converges asymptotically. This significantly slows down the convergence of the eigenvalue estimation. To avoid this, we propose a different method in which normalization is done locally, at each node, without inter-node communication. In this way, the optimal scaling parameter can be obtained using a distributed method. We then address the problem of designing the preconditioning matrix. Our distributed scenario constrains us to use a block diagonal matrix. Choosing the optimal matrix under this constraint, and using only distributed processing, is a very challenging problem for which we are not able to provide a solution. Nevertheless, we are able to bound the difference between the convergence rate achieved using the optimal matrix, and the convergence rate resulting using a practically feasible matrix design. This bound turns out to have a simple expression which depends on the network connectivity. A shortened version of this method appears in the conference paper (Marelli & Fu, 2013).

For an acyclic network (i.e., its communication graph contains no loops), we provide a different iterative algorithm for distributed estimation. As opposed to the previous algorithm, in this one, the composite estimate is guaranteed to converge to the globally optimal estimate in a finite number of steps. Indeed, we show that the convergence time equals the diameter of the aforementioned graph. Numerical experiments show that this method offers a major reduction in convergence time.

The rest of paper is organized as follows. In Section  2 we describe the distributed WLS estimation the problem. In Section  3 we derive the first distributed WLS method, which converges asymptotically. In Section  3.1 we describe distributed methods for finding the value of the scaling parameter which yields the fastest convergence rate. In Section  3.2 we describe a sub-optimal choice for preconditioning matrix, and we bound its sub-optimality. In Section  4 we introduce the second distributed WLS method which converges in finite time. Numerical experiments are presented in Section  5, and concluding remarks are given in Section  6. For the ease of readability, all proofs are contained in the Appendix A, Appendix B.

Notation 1 *For a vector*x*,*‖x‖*denotes its*  2*-norm, and* ${\left[x\right]}_{i}$ *denotes its* ith *entry. For a matrix* X*,* ‖X‖ *denotes its operator (induced) norm. For square symmetric matrices* X *and* Y*,* X<Y *means that matrix* Y−X *is positive-definite. For vectors and matrices, the superscript* ${}^{T}$ *denotes its transpose, and* ${}^{*}$ *denotes its transpose conjugate.*

## 2 Problem description

Consider a network formed by I nodes. For each i=1,…,I, Node i has an associated parameter vector $x_{i}\in C^{d_{i}}$, and measures the vector $y_{i}\in C^{p_{i}}$, which is given by

(1) $$y_{i}=\overset{I}{\underset{j=1}{\sum }}A_{i,j}x_{j}+v_{i}\text{,}$$

where $v_{i}∼N\left(0,R_{i}\right)$ denotes the measurement noise. The noises $v_{i}$ and $v_{j}$ are statistically independent, whenever i≠j.

Let $x^{T}=\left[x_{1}^{T},\ldots ,x_{I}^{T}\right]$, $y^{T}=\left[y_{1}^{T},\ldots ,y_{I}^{T}\right]$, $v^{T}=\left[v_{1}^{T},\ldots ,v_{I}^{T}\right]$, $R=\mathrm{diag}\left\{R_{1},\ldots ,R_{I}\right\}$ and $A={\left[A_{i,j}\right]}_{i,j=1,\ldots ,I}$. Then, we can write the measurement model of the whole network as

(2) y=Ax+v,

with v∼N(0,R). The WLS estimator $\overset{ˆ}{x}$ of x is defined by Kay (1993, Eq. (8.14))

$$\overset{ˆ}{x}=\arg\underset{x}{\min}{\left(y-Ax\right)}^{*}R^{-1}\left(y-Ax\right)\text{.}$$

Its solution is given by

(3) $$\overset{ˆ}{x}=\Psi^{-1}\alpha$$

with

$$\alpha =A^{*}R^{-1}y\;\text{and}\;\Psi =A^{*}R^{-1}A\text{.}$$

For the WLS problem to be well defined, we make the following assumption:

Assumption 2 Matrix A has full column rank and R is non-singular.

Computing (3) requires global information, i.e., all the measurements and the information about A and R need to be made available together. Our goal is to derive distributed methods in which Node i computes the component ${\overset{ˆ}{x}}_{i}$ of the estimate $\overset{ˆ}{x}$, corresponding to $x_{i}$, using only the local measurement $y_{i}$ and information received from its neighbors (a formal definition of neighborhood will be given later).

In the rest of the paper we use the following notation:

Notation 3 *Let*$I_{i}=\left\{j:A_{j,i}\neq 0\right\}$*denote the set of nodes whose measurements involve the parameters of Node*i*, and*$O_{i}=\left\{j:A_{i,j}\neq 0\right\}$*denote the set of nodes whose parameters are involved in the measurements of Node*i*. Let*$N_{i}=I_{i}\cup O_{i}$*be the *neighborhood* of Node*i*. We also define*$B_{i}=\left\{j:N_{i}\cap N_{j}\neq 0̸\right\}$*to be the set of nodes whose neighborhood have a non-empty intersection with that of Node*i*. Notice that*$B_{i}=\left\{j:\Psi_{i,j}\neq 0\right\}$*.*

## 3 Asymptotic method for WLS estimation

The distributed WLS method derived in this section uses the following definition of neighbor node.

Definition 4 Node j is a neighbor of Node i if $j\in N_{i}$.

Also, the proposed method requires the following connectivity assumption:

Assumption 5 For each i=1,…,I, Node i can send/receive information to/from all its neighbors. Also, $A_{i,j}$, for all $j\in O_{i}$, and $R_{i}$ are available at Node i.

Although our method works regardless of whether the network is sparse or not, it works most efficiently for sparse networks. A network is called sparse if the cardinality of $N_{i}$ is small for all i=1,2,…,I.

Consider any block diagonal positive definite matrix

(4) $$\Pi =\mathrm{diag}\left\{\Pi_{1},\ldots ,\Pi_{I}\right\}$$

with $\Pi_{i}\in C^{d_{i}\times d_{i}},i=1,\ldots ,I$. Then define

(5) $$\Upsilon =\Pi^{1/2}\Psi \Pi^{1/2}$$

and choose

(6) $$0<\gamma <2{\left\|\Upsilon \right\|}^{-1}\text{.}$$

Let $\overset{\sim }{\alpha }={\left(\gamma \Pi \right)}^{1/2}\alpha$ and $\overset{\sim }{\overset{ˆ}{x}}={\left(\gamma \Pi \right)}^{-1/2}\overset{ˆ}{x}$. From (3) we have

$$\overset{\sim }{\overset{ˆ}{x}}={\left(\gamma \Upsilon \right)}^{-1}\overset{\sim }{\alpha }\text{.}$$

From (6), 0<γΥ<2I. Hence, −I<γΥ−I<I and therefore ‖I−γΥ‖<1. In view of this, we can use Richardson’s method (Bertsekas & Tsitsiklis, 1997) to compute $\overset{\sim }{\overset{ˆ}{x}}$ recursively. This leads to

(7) $$\overset{\sim }{\overset{ˆ}{x}}\left(t+1\right)=\left(I-\gamma \Upsilon \right)\overset{\sim }{\overset{ˆ}{x}}\left(t\right)+\overset{\sim }{\alpha }\text{.}$$

Then, by substituting the expressions of $\overset{\sim }{\alpha }$ and $\overset{\sim }{\overset{ˆ}{x}}$, we obtain straightforwardly

(8) $$\overset{ˆ}{x}\left(t+1\right)=\left(I-\gamma \Pi \Psi \right)\overset{ˆ}{x}\left(t\right)+\gamma \Pi \alpha \text{.}$$

We call Π the preconditioning matrix, because, as it will be explained in Section  3.2, it is used to increase the convergence rate of the recursions (8).

Let

(9) $$\alpha_{i}=\underset{k\in I_{i}}{\sum }\overset{\left(k\right)}{\underset{i}{\alpha }}\text{,}$$

with $\alpha_{i}^{\left(k\right)}=A_{k,i}^{*}R_{k}^{-1}y_{k}$, for k=1,…,I, so that $\alpha^{T}=\left[\alpha_{1}^{T},\ldots ,\alpha_{I}^{T}\right]$. Also, for i,j=1,…,I, let

(10) $$\Psi_{i,j}=\underset{k:i,j\in O_{k}}{\sum }\overset{\left(k\right)}{\underset{i,j}{\Psi }}\text{,}$$

where $\Psi_{i,j}^{\left(k\right)}=A_{k,i}^{*}R_{k}^{-1}A_{k,j}$, for all k=1,…,I, so that $\Psi ={\left[\Psi_{i,j}\right]}_{i,j=1,\ldots ,I}$. We have that

(11) $${\left[\Psi \overset{ˆ}{x}\left(t\right)\right]}_{i}=\overset{I}{\underset{j=1}{\sum }}\Psi_{i,j}{\overset{ˆ}{x}}_{j}\left(t\right)=\overset{I}{\underset{j=1}{\sum }}\underset{k:i,j\in O_{k}}{\sum }\overset{\left(k\right)}{\underset{i,j}{\Psi }}{\overset{ˆ}{x}}_{j}\left(t\right)=\underset{k:i\in O_{k}}{\sum }\underset{j\in O_{k}}{\sum }\overset{\left(k\right)}{\underset{i,j}{\Psi }}{\overset{ˆ}{x}}_{j}\left(t\right)=\underset{k\in I_{i}}{\sum }\underset{j\in O_{k}}{\sum }\overset{\left(k\right)}{\underset{i,j}{\Psi }}{\overset{ˆ}{x}}_{j}\left(t\right)\text{.}$$

Then, from (8), (11), (9), we obtain

(12) $${\overset{ˆ}{x}}_{i}\left(t+1\right)={\overset{ˆ}{x}}_{i}\left(t\right)-\gamma \Pi_{i}\overset{I}{\underset{j=1}{\sum }}\Psi_{i,j}{\overset{ˆ}{x}}_{j}\left(t\right)+\gamma \Pi_{i}\alpha_{i}={\overset{ˆ}{x}}_{i}\left(t\right)-\gamma \Pi_{i}\left(\underset{k\in I_{i}}{\sum }\underset{j\in O_{k}}{\sum }\overset{\left(k\right)}{\underset{i,j}{\Psi }}{\overset{ˆ}{x}}_{j}\left(t\right)-\underset{k\in I_{i}}{\sum }\overset{\left(k\right)}{\underset{i}{\alpha }}\right)\text{.}$$

Notice that the matrices $\Psi_{i,j}^{\left(k\right)}$ are only available at Node k. That is, Node k acts as an intermediary between Node j, which transmits ${\overset{ˆ}{x}}_{j}\left(t\right)$, and Node i, which receives $\sum_{j\in O_{k}}\Psi_{i,j}^{\left(k\right)}{\overset{ˆ}{x}}_{j}\left(t\right)$. This means that node  j should transmit ${\overset{ˆ}{x}}_{j}\left(t\right)$ to all nodes k with $j\in O_{k}$, or equivalently, to all nodes in $I_{j}$. However, Node j does not know which nodes are in $I_{j}$. Thus, Node j simply transmits ${\overset{ˆ}{x}}_{j}\left(t\right)$ to all nodes in $N_{j}$, and it is up to the receiving Node k to detect whether Node $j\in O_{k}$ or not.

Following the discussion above, we obtain the following algorithm to implement (12).

**Algorithm 1 - distributed WLS estimation**:

**Initialization:**

(1) For each k=1,⋯,I and $i\in O_{k}$, Node  k computes $\alpha_{i}^{\left(k\right)}$ and sends it to Node  i.
(2) On reception, Node  i computes $\alpha_{i}=\sum_{k\in I_{i}}\alpha_{i}^{\left(k\right)}$.
(3) For each i=1,⋯,I, Node  i sets ${\overset{ˆ}{x}}_{i}\left(1\right)=0$.

**Main loop:**  At time t∈N:

(1) For each j=1,⋯,I and $k\in N_{j}$, Node  j sends its current estimate ${\overset{ˆ}{x}}_{j}\left(t\right)$ to Node  k.
(2) On reception, for each k=1,⋯,I and $i\in O_{k}$, Node  k sends to Node  i
  $${\overset{ˇ}{x}}_{i,k}\left(t\right)=\underset{j\in O_{k}}{\sum }\overset{\left(k\right)}{\underset{i,j}{\Psi }}{\overset{ˆ}{x}}_{j}\left(t\right)\text{.}$$
(3) On reception, for each i=1,⋯,I, Node  i computes
  $${\overset{ˆ}{x}}_{i}\left(t+1\right)={\overset{ˆ}{x}}_{i}\left(t\right)-\gamma \Pi_{i}\left(\underset{k\in I_{i}}{\sum }{\overset{ˇ}{x}}_{i,k}\left(t\right)-\alpha_{i}\right)\text{.}$$

To implement Algorithm 1, we need to design the scaling factor γ and the preconditioning matrices $\Pi_{i}$, for all i=1,…,I. We address these two tasks in Sections  3.1, 3.2, respectively.

### 3.1 Distributed design of the scaling factor γ

In this section we study two approaches for designing the scaling factor γ. In Section  3.1.1 we describe a distributed algorithm which converges asymptotically to the optimal value of γ, i.e., the resulting γ will achieve the maximum convergence speed. In Section  3.1.2, we give another distributed algorithm which converges in finite time to a sub-optimal value of γ.

#### 3.1.1 Asymptotic algorithm for γ

In view of (7), the value of γ that maximizes the convergence rate is

(13) $$\gamma =\frac{2}{\left\|\Upsilon \right\|+{\left\|\Upsilon^{-1}\right\|}^{-1}}\text{,}$$

because this is the value that minimizes ‖I−γΥ‖. In order for each node to find the value of γ given in (13), we need distributed methods for finding ‖Υ‖ and ${\left\|\Upsilon^{-1}\right\|}^{-1}$. We give these methods below. These methods yield, at Node i and time step t, estimates ${\bar{\Upsilon }}_{i}\left(t\right)$ and ${\underline{\Upsilon }}_{i}\left(t\right)$, of ‖Υ‖ and ${\left\|\Upsilon^{-1}\right\|}^{-1}$, respectively. Then, at the same node and time step, the estimate $\gamma_{i}\left(t\right)$ of γ is obtained by

$$\gamma_{i}\left(t\right)=\frac{2}{{\bar{\Upsilon }}_{i}\left(t\right)+{\underline{\Upsilon }}_{i}\left(t\right)}\text{.}$$

**Distributed method for finding**‖Υ‖:

Choose any vector b(0)≠0 and let $b\left(t\right)=\Upsilon^{t}b\left(0\right)$. Then, using (11) with $\Pi^{1/2}b\left(t\right)$ in place of $\overset{ˆ}{x}\left(t\right)$, we obtain at Node i,

(14) $$b_{i}\left(t+1\right)={\left[\Upsilon b\left(t\right)\right]}_{i}=\Pi_{i}^{1/2}{\left[\Psi \Pi^{1/2}b\left(t\right)\right]}_{i}=\Pi_{i}^{1/2}\underset{k\in I_{i}}{\sum }\underset{j\in O_{k}}{\sum }\overset{\left(k\right)}{\underset{i,j}{\Psi }}\overset{1/2}{\underset{j}{\Pi }}b_{j}\left(t\right)\text{,}$$

where $b_{i}\left(t\right)$ denotes the ith block component of b(t). Then, using the power method (Bertsekas & Tsitsiklis, 1997), Node i can asymptotically compute ‖Υ‖ as follows

(15) $$\left\|\Upsilon \right\|=\underset{t\to \infty }{\lim}\frac{\left\|b_{i}\left(t\right)\right\|}{\left\|b_{i}\left(t-1\right)\right\|}\text{.}$$

An inconvenience with the approach above is that b(t) either increases or decreases indefinitely. To avoid this, the vector b(t) can be periodically normalized. In Yang et al. (2010), this was done using average consensus (in the continuous-time case). As we mentioned in Section  1, we avoid the drawbacks of that method by providing an alternative approach in which b(t) is normalized at each node, and each iteration, without inter-node communication. This algorithm is given below:

**Algorithm 2 - distributed estimation** of ‖Υ‖: For each k=1,⋯,I, Node  k, chooses ${\overset{¯}{b}}_{k}\left(1\right)$, with $\left\|{\overset{¯}{b}}_{k}\left(1\right)\right\|=1$ and sets $\varsigma_{k}\left(1\right)=1$ and $\upsilon_{i,j}^{\left(k\right)}\left(1\right)=1$, for all $i,j\in N_{k}$. Then, at time t∈N:

(1) For each j=1,⋯,I and $k\in N_{j}$, Node  j sends $\left(\Pi_{j}^{1/2}{\overset{¯}{b}}_{j}\left(t\right),\varsigma_{j}\left(t\right)\right)$ to Node  k.
(2) On reception, for each k=1,⋯,I and $i\in O_{k}$, Node  k sends $\left({\overset{ˇ}{b}}_{i}^{\left(k\right)}\left(t\right),{\overset{¯}{\varsigma }}_{i}^{\left(k\right)}\left(t\right)\right)$ to Node  i, where
  $${\overset{ˇ}{b}}_{i}^{\left(k\right)}\left(t\right)=\underset{j\in O_{k}}{\sum }\overset{\left(k\right)}{\underset{i,j}{\upsilon }}\left(t\right)\Psi_{i,j}^{\left(k\right)}\Pi_{j}^{1/2}{\overset{¯}{b}}_{j}\left(t\right),{\overset{¯}{\varsigma }}_{i}^{\left(k\right)}\left(t\right)=\underset{j\in N_{k}}{\max}\overset{\left(k\right)}{\underset{i,j}{\upsilon }}\left(t\right)\text{,}$$
  and
  $$\upsilon_{i,j}^{\left(k\right)}\left(t\right)=\frac{\varsigma_{i}\left(t\right)}{\varsigma_{j}\left(t\right)}\upsilon_{i,j}^{\left(k\right)}\left(t-1\right)\text{.}$$
(3) On reception, for each i=1,⋯,I, Node  i computes
  $${\overset{¯}{b}}_{i}\left(t+1\right)=\varsigma_{i}\left(t+1\right){\overset{\sim }{b}}_{i}\left(t+1\right),\varsigma_{i}\left(t+1\right)=\max{\left\{\left\|{\overset{\sim }{b}}_{i}\left(t+1\right)\right\|,\overset{\left(k\right)}{\underset{i}{\overset{¯}{\varsigma }}}\left(t\right),k\in I_{i}\right\}}^{-1}\text{,}$$
  with
  (16) $${\overset{\sim }{b}}_{i}\left(t+1\right)=\Pi_{i}^{1/2}\underset{k\in I_{i}}{\sum }\overset{\left(k\right)}{\underset{i}{\overset{ˇ}{b}}}\left(t\right)\text{.}$$
  Also, the estimate ${\bar{\Upsilon }}_{i}\left(t\right)$ of ‖Υ‖ is
  (17) $${\bar{\Upsilon }}_{i}\left(t\right)=\varsigma_{i}{\left(t+1\right)}^{-1}\text{.}$$

The convergence of Algorithm 2 to ‖Υ‖ is guaranteed by the next theorem.

Theorem 6 *Consider the network*   (1)   *together with*   Assumption 2, Assumption 5*. Then, for each* i∈{1,…,I}*,*

(18) $$\underset{t\to \infty }{\lim}{\bar{\Upsilon }}_{i}\left(t\right)=\left\|\Upsilon \right\|\text{.}$$

**Distributed method for finding**${\left\|\Upsilon^{-1}\right\|}^{-1}$:

Let c≥‖Υ‖ and Φ=cI−Υ. It follows that

$${\left\|\Upsilon^{-1}\right\|}^{-1}=\underline{\mathrm{eig}}\left(\Upsilon \right)=c-\bar{\mathrm{eig}}\left(\Phi \right)=c-\left\|\Phi \right\|\text{,}$$

where, for a symmetric matrix X, $\underline{\mathrm{eig}}\left(X\right)$ and $\bar{\mathrm{eig}}\left(X\right)$ denote the smallest and largest eigenvalues of X, respectively. Hence, we can find ${\left\|\Upsilon^{-1}\right\|}^{-1}$ by applying Algorithm 2 on Φ, to find ‖Φ‖. To this end, at Node i and time t, we choose $c={\bar{\Upsilon }}_{i}\left(t\right)$. This leads to the following algorithm:

**Algorithm 3 - distributed estimation** of ${\left\|\Upsilon^{-1}\right\|}^{-1}$: Apply Algorithm 2 with (16) replaced by

$${\overset{\sim }{b}}_{i}\left(t+1\right)={\bar{\Upsilon }}_{i}\left(t\right){\overset{¯}{b}}_{i}\left(t\right)-\Pi_{i}^{1/2}\underset{k\in I_{i}}{\sum }{\overset{ˇ}{b}}_{i,k}\left(t\right)\text{,}$$

and  (17) replaced by

$${\underline{\Upsilon }}_{i}\left(t\right)={\bar{\Upsilon }}_{i}\left(t\right)-{\bar{\Phi }}_{i}\left(t\right),{\bar{\Phi }}_{i}\left(t\right)=\varsigma_{i}{\left(t+1\right)}^{-1}\text{.}$$

#### 3.1.2 Finite-time algorithm for γ

A sub-optimal design of γ can be achieved using the following result.

Theorem 7 *Condition*   (6)   *is satisfied by choosing* γ *so that*

$$0<\gamma <\frac{2}{\underset{i}{\max}\phi_{i}}\text{,}$$

*where*

$$\phi_{i}=\underset{k\in I_{i}}{\sum }\upsilon_{i,k}\text{,}$$

$$\upsilon_{i,k}=\underset{j\in O_{k}}{\sum }\left\|\overset{1/2}{\underset{i}{\Pi }}\Psi_{i,j}^{\left(k\right)}\Pi_{j}^{1/2}\right\|\text{.}$$

The design of γ using Theorem 7 requires the global information $\max_{i}\phi_{i}$. For each, i=1,…,I, Node i can obtain $\phi_{i}$ from an initialization stage in which it receives $\upsilon_{i,k}$, from each Node k, with $k\in I_{i}$. Then, $\max_{i}\phi_{i}$ can be obtained by running the max-consensus algorithm (Olfati-Saber & Murray, 2004), in parallel with the estimation Algorithm 1. Notice that the max-consensus algorithm converges in finite time.

### 3.2 Design of the preconditioning matrix Π

As mentioned above, for a given choice of Υ, the fastest convergence rate of Algorithm 1 is achieved when γ is chosen as in (13). Under this choice of γ, we have that

$$\left\|I-\gamma \Upsilon \right\|=\gamma \left\|\Upsilon \right\|-1=\frac{2\left\|\Upsilon \right\|}{\left\|\Upsilon \right\|+{\left\|\Upsilon^{-1}\right\|}^{-1}}-1=\frac{\left\|\Upsilon \right\|-{\left\|\Upsilon^{-1}\right\|}^{-1}}{\left\|\Upsilon \right\|+{\left\|\Upsilon^{-1}\right\|}^{-1}}=\frac{\kappa \left(\Upsilon \right)-1}{\kappa \left(\Upsilon \right)+1}\text{,}$$

where $\kappa \left(\Upsilon \right)=\left\|\Upsilon \right\|\left\|\Upsilon^{-1}\right\|$ denotes the condition number of Υ. Then, from (7), there exists K≥0, such that

$$\left\|\overset{ˆ}{x}-\overset{ˆ}{x}\left(t\right)\right\|\leq K{\left\|I-\gamma \Upsilon \right\|}^{t}=K\exp\left\{t\log\frac{\kappa \left(\Upsilon \right)-1}{\kappa \left(\Upsilon \right)+1}\right\}\text{,}$$

where we recall that $\overset{ˆ}{x}$ denotes the global estimate of x, given by (3). Then, we define the time constant τ(Υ) of the distributed WLS algorithm by

(19) $$\tau \left(\Upsilon \right)=\frac{1}{\log\frac{\kappa \left(\Upsilon \right)+1}{\kappa \left(\Upsilon \right)-1}}\text{.}$$

Hence, a natural question is whether the preconditioning matrices $\Pi_{i}$, i=1,…,I, can be chosen so that τ(Υ) is minimized. While we are not able to answer this question, we have the following result, which follows using an argument similar to the one in Demmel (1983, Theorem 2).

Theorem 8 *If*$\Pi_{i}=\Psi_{i,i}^{-1}$*, for all*i=1,…,I*, then*

$$\kappa \left(\Upsilon \right)\leq \beta \kappa_{⋆}\text{,}$$

*where*

$$\beta =\underset{i}{\max}\left|B_{i}\right|\text{,}$$

$$\kappa_{⋆}=\underset{\overset{\sim }{\Pi }\in P}{\min}\kappa \left({\overset{\sim }{\Pi }}^{1/2}\Psi {\overset{\sim }{\Pi }}^{1/2}\right)\text{,}$$

 with  P *denoting the set of positive definite block diagonal matrices of the form*   (4)*.*

Theorem 8 states that, if the preconditioning matrices $\Pi_{i}$, i=1,…,I, are chosen as

(20) $$\Pi_{i}=\Psi_{i,i}^{-1}$$

then κ(Υ) is at most β times bigger than the smallest possible value $\kappa_{⋆}$ achievable using block diagonal preconditioning matrices. Notice that $B_{i}=\left\{j:I_{i}\cap I_{j}\neq 0̸\right\}\subseteq \left\{j:N_{i}\cap N_{j}\neq 0̸\right\}$. Hence, β is bounded by the maximum number of two-hop neighbors over the whole network. Hence, it does not necessarily grow with the network size.

Now, we have

$$\underset{\kappa \to \infty }{\lim}\kappa \log\left(\frac{\kappa +1}{\kappa -1}\right)=2\text{.}$$

Hence, from Theorem 8, for large κ(Υ), we have

(21) $$\tau \left(\Upsilon \right)≃\frac{\kappa \left(\Upsilon \right)}{2}\leq \frac{\beta }{2}\underset{\overset{\sim }{\Pi }}{\min}\kappa \left({\overset{\sim }{\Pi }}^{1/2}\Psi {\overset{\sim }{\Pi }}^{1/2}\right)≃\beta \tau_{⋆}\text{,}$$

where

$$\tau_{⋆}=\underset{\overset{\sim }{\Pi }\in P}{\min}\tau \left({\overset{\sim }{\Pi }}^{1/2}\Psi {\overset{\sim }{\Pi }}^{1/2}\right)\text{.}$$

Hence, if $\Pi_{i}$, i=1,…,I, are chosen as in (20), and κ(Υ) is large, then the time constant τ(Υ) is at most β times bigger than the minimum value $\tau_{⋆}$.

Remark 9 In view of (20), (10),

$$\Pi_{i}={\left(\underset{k\in I_{i}}{\sum }\overset{\left(k\right)}{\underset{i,i}{\Psi }}\right)}^{-1}\text{.}$$

Hence, its computation requires the matrices $\Psi_{i,i}^{\left(k\right)}$, $k\in I_{i}$, to be transmitted from Node k to Node i during an initialization stage.

## 4 Finite-time method for WLS estimation

In this method we replace the definition of neighborhood by the following one:

Definition 10 Node j is a neighbor of Node i if $j\in B_{i}$.

Consequently, we replace the connectivity Assumption 5 by the following one:

Assumption 11 For each i=1,…,I, Node i can send/receive information to/from all its neighbors. Also, $\Psi_{j,i}$, for all $j\in B_{i}$, and $\alpha_{i}$ are available at Node i.

To illustrate the idea behind the proposed algorithm, we consider a network with two nodes. The next lemma states how to obtain the global optimal solution, at each node, in this simple case.

Lemma 12 *Consider the network*   (1)   *together with*   Assumption  2*. If there are only two nodes, labeled by* a *and* b*, then* $\Psi_{a,a}-\Psi_{a,b}{\overset{ˇ}{\Sigma }}_{b}\Psi_{b,a}$ *is an invertible matrix and the global estimate* ${\overset{ˆ}{x}}_{a}$ *of the components* $x_{a}$ *associated to Node* a *is given by*

$${\overset{ˆ}{x}}_{a}=\Sigma_{a}\left(\alpha_{a}-\Psi_{a,b}{\overset{ˇ}{x}}_{b}\right)\text{,}$$

$$\Sigma_{a}={\left(\Psi_{a,a}-\Psi_{a,b}{\overset{ˇ}{\Sigma }}_{b}\Psi_{b,a}\right)}^{-1}\text{,}$$

*where*

$${\overset{ˇ}{x}}_{b}={\overset{ˇ}{\Sigma }}_{b}\alpha_{b}\text{,}$$

$${\overset{ˇ}{\Sigma }}_{b}=\Psi_{b,b}^{-1}\text{.}$$

Our next result is an immediate generalization of the one above, to a network with a star topology, i.e., in which all nodes are only possibly connected to a single one.

Lemma 13 *Consider the network*   (1)   *together with*   Assumption  2*. Suppose that* $\Psi_{j,k}=0$*, for all* j,k∈{1,…,I}∖{i} *and* j≠k *(i.e., all nodes are only possibly connected to Node* i *). Then* $\Psi_{i,i}-\sum_{j\in B_{i}\setminus \left\{i\right\}}\Psi_{i,j}{\overset{ˇ}{\Sigma }}_{j}\Psi_{j,i}$ *is an invertible matrix and* ${\overset{ˆ}{x}}_{i}$ *is given by*

$${\overset{ˆ}{x}}_{i}=\Sigma_{i}\left(\alpha_{i}-\underset{j\in B_{i}\setminus \left\{i\right\}}{\sum }\Psi_{i,j}{\overset{ˇ}{x}}_{j}\right)\text{,}$$

$$\Sigma_{i}={\left(\Psi_{i,i}-\underset{j\in B_{i}\setminus \left\{i\right\}}{\sum }\Psi_{i,j}{\overset{ˇ}{\Sigma }}_{j}\Psi_{j,i}\right)}^{-1}\text{,}$$

*where*

$${\overset{ˇ}{x}}_{j}={\overset{ˇ}{\Sigma }}_{j}\alpha_{j}\text{,}$$

$${\overset{ˇ}{\Sigma }}_{j}=\Psi_{j,j}^{-1}\text{.}$$

Then, using (11), (9) and Lemma 13, we obtain the following algorithm:

**Algorithm 4 - distributed WLS estimation**:

**Initialization:** For each i=1,⋯,I,

(1) Node  i computes
  $${\overset{ˇ}{x}}_{i}\left(0\right)={\overset{ˇ}{\Sigma }}_{i}\left(0\right)\alpha_{i},{\overset{ˇ}{\Sigma }}_{i}\left(0\right)=\Psi_{i,i}^{-1}\text{.}$$
  and for each $j\in B_{i}\setminus \left\{i\right\}$,
  $${\overset{ˇ}{x}}_{i,j}\left(0\right)={\overset{ˇ}{x}}_{i}\left(0\right),{\overset{ˇ}{\Sigma }}_{i,j}\left(0\right)={\overset{ˇ}{\Sigma }}_{i}\left(0\right)\text{.}$$

**Main loop:** For each i=1,⋯,I, and time t∈N

(1) Node  i computes, for each $j\in B_{i}\setminus \left\{i\right\}$,
  $$\gamma_{i,j}\left(t\right)=\Psi_{j,i}{\overset{ˇ}{x}}_{i}\left(t-1\right),\Gamma_{i,j}\left(t\right)=\Psi_{j,i}{\overset{ˇ}{\Sigma }}_{i}\left(t-1\right)\Psi_{i,j}\text{,}$$
  and sends $\left(\gamma_{i,j}\left(t\right),\Gamma_{i,j}\left(t\right)\right)$ to Node  j.
(2) Node  i computes
  $${\overset{ˇ}{x}}_{i}\left(t\right)={\overset{ˇ}{\Sigma }}_{i}\left(t\right)\left(\alpha_{i}-\underset{j\in B_{i}\setminus \left\{i\right\}}{\sum }\gamma_{j,i}\left(t-1\right)\right),{\overset{ˇ}{\Sigma }}_{i}\left(t\right)={\left(\Psi_{i,i}-\underset{j\in B_{i}\setminus \left\{i\right\}}{\sum }\Gamma_{j,i}\left(t-1\right)\right)}^{-1}\text{,}$$
  and, for each $j\in B_{i}\setminus \left\{i\right\}$,
  $${\overset{ˇ}{x}}_{i,j}\left(t\right)={\overset{ˇ}{\Sigma }}_{i,j}\left(t\right)\left(\alpha_{i}-\underset{j\in B_{i}\setminus \left\{i,j\right\}}{\sum }\gamma_{j,i}\left(t-1\right)\right),{\overset{ˇ}{\Sigma }}_{i,j}\left(t\right)={\left(\Psi_{i,i}-\underset{j\in B_{i}\setminus \left\{i,j\right\}}{\sum }\Gamma_{j,i}\left(t-1\right)\right)}^{-1}\text{.}$$

Our next step is to show that Algorithm 4 converges in finite time to the global WLS solution.

Definition 14 Each pair (i,j), i,j∈{1,…,I}, is called an *edge* if $\Psi_{i,j}\neq 0$. A *path* is a concatenation of contiguous edges, and its *length* is the number of edges forming it. For each i,j∈{1,…,I}, the *distance* $d_{i,j}$ between Nodes i and j is defined as the minimum length of a path joining these two nodes. The *radius* $\rho_{i}$ of Node i is defined as the maximum distance between Node i and any other node in the network. The *diameter* of the network is the maximum radius between all its nodes. A network is called *acyclic* if it does not contain a path forming a cycle.

The next theorem states that, if the network is acyclic, then the algorithm above yields the global estimate at each node in finite time.

Theorem 15 *Consider the network*   (1)   *together with*   Assumption 2, Assumption 11*. If the network is acyclic, then, for each* i∈{1,…,I}*,* $j\in B_{i}\setminus \left\{i\right\}$ *and* t∈N*, the matrices* $\Psi_{i,i}-\sum_{j\in B_{i}\setminus \left\{i\right\}}\Gamma_{j,i}\left(t-1\right)$ *and* $\Psi_{i,i}-\sum_{j\in B_{i}\setminus \left\{i,j\right\}}\Gamma_{j,i}\left(t-1\right)$ *are invertible, and for all* $t\geq \rho_{i}$*,*

(22) $${\overset{ˇ}{x}}_{i}\left(t\right)={\overset{ˆ}{x}}_{i}\text{.}$$

## 5 Simulations

### 5.1 State estimation in power systems

**Fig. 1:**
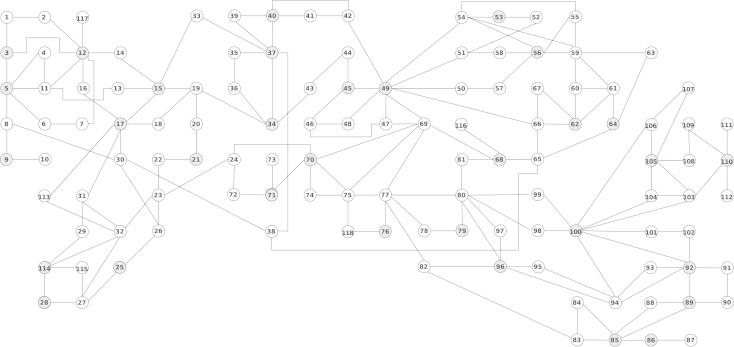
Diagram of the IEEE 118-bus test system.

In the first simulation we use the proposed distributed methods for state estimation in smart electricity networks, involving multi-area interconnected power systems (Huang et al., 2012). To this end, we use the IEEE 118-bus test system, whose specifications are given in Christie (1993). The system’s diagram is shown in Fig. 1, where buses are represented by circles and lines by edges. Some buses have a phasor measurement unit (PMU) installed. These buses are shown in gray. Each PMU measures the voltage of the bus where it is installed, as well as the currents of the lines attached to that bus. The goal is to estimate the state vector x, containing voltage (a complex phasor) at each bus. For the purposes of state estimation, the buses are clustered in nodes. Two clustering examples as shown in Table 1, Table 3.

**Table 1:** Nodes forming a cyclic network topology.

Node	Buses
1	1, 2, 3, 4, 5, 6, 7, 8, 9, 10, 11, 12, 14, 16, 117
2	13, 15, 17, 18, 19, 20, 21, 22, 23, 25, 26, 27, 28, 29, 30, 31, 32, 33, 113, 114, 115
3	24, 38, 70, 71, 72, 73, 74
4	34, 35, 36, 37, 39, 40, 41, 42, 43
5	44, 45, 46, 47, 48, 49, 50, 51, 52, 53, 54, 55, 56, 57, 58, 59, 60, 61, 62, 63, 64, 65, 66, 67, 68, 69, 77, 80, 81, 100, 116
6	75, 76, 78, 79, 82, 95, 96, 97, 98, 118
7	83, 84, 85, 86, 87, 88, 89, 90, 91, 92, 93, 94
8	99, 101, 102, 103, 104, 105, 106, 107, 108, 109, 110, 111, 112

Let P denote the number of PMUs in the whole system. For each p=1,…,P, let $L_{p}$ denote the number of lines attached to the bus where PMU  p is installed. Let also $B_{p}^{T}=\left[e_{p}^{T},y_{p,1}^{T},\ldots ,y_{p,L_{p}}^{T}\right]$, where the vectors $e_{p}$ and $y_{p,l}$, $l=1,\ldots ,L_{p}$, are defined such that $e_{p}x$ is the voltage of the installation bus, and $y_{p,l}x$ is the current of the p-th attached line (the value of $y_{p,l}$ is taken from Christie, 1993). Then, matrix A in (2) is given by $A^{T}=\left[A_{1}^{T},\ldots ,A_{I}^{T}\right]$, where, for each i=1,…,I, the block of rows $A_{i}$ corresponding to Node i is formed by stacking the matrices $B_{p}$ corresponding to all PMUs contained in Node i, i.e., $A_{i}^{T}=\left[B_{p_{1}}^{T},\ldots ,B_{p_{P_{i}}}^{T}\right]$, where $p_{1},\ldots ,p_{P_{i}}$ denote the indexes of those PMUs.

We place the PMUs using the method in Tai et al. (2013). This guarantees that matrix A has full column rank. We also assume that the noise covariance is $R=\sigma^{2}I$, with σ=0.05. Notice that voltage and current values in the test system are *per unit* values, i.e., they appear divided by the nominal voltage $V_{0}$ and the nominal current $I_{0}$, respectively. Hence, σ=0.05 means that voltage measurements have a standard deviation of $0.05\times V_{0}$ volts, and current measurements have one of $0.05\times I_{0}$ amperes. This leads to a global estimate $\overset{ˆ}{x}$ having a relative estimation error of

(23) $$e=20\log_{10}\frac{\left\|x-\overset{ˆ}{x}\right\|}{\left\|x\right\|}=-17.45\;\text{dB .}$$

In the simulations below, we use

(24) $$r\left(t\right)=20\log_{10}\frac{\left\|\overset{ˆ}{x}-\overset{ˆ}{x}\left(t\right)\right\|}{\left\|\overset{ˆ}{x}\right\|}\text{,}$$

to measure the relative difference between the global estimate $\overset{ˆ}{x}$ and the one yielded, at time step t, by the proposed distributed algorithms.

#### 5.1.1 Cyclic network topology

**Fig. 2:**
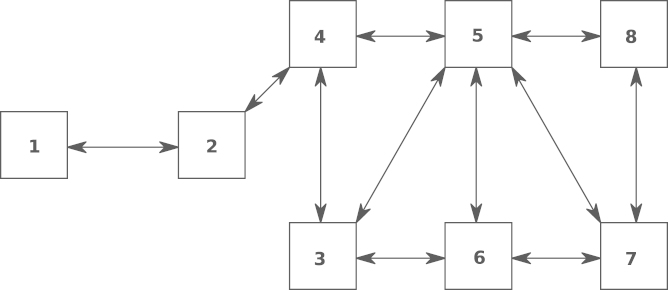
Cyclic network topology induced by the nodes in Table 1.

In the first simulation we cluster the buses into eight nodes, as shown in Table 1. From the definition of $N_{i}$, it follows that $j\in N_{i}$ if there is a bus in either, Node i or j, with a PMU installed, having an attached line coming from a bus inside the other node. Fig. 2 shows the topology of the communication network induced by the clustering given in Table 1.

**Fig. 3:**
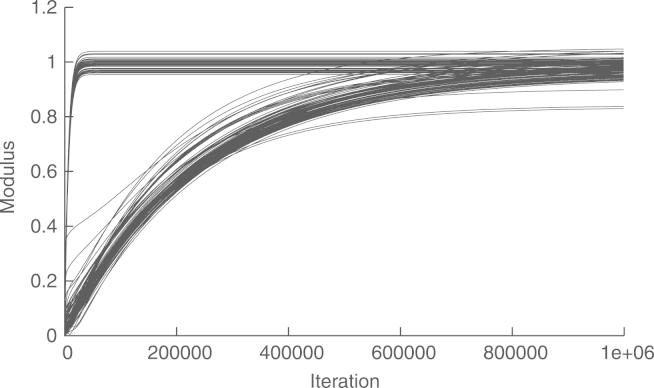
Convergence of the asymptotic method, without preconditioning, in a cyclic network.

**Fig. 4:**
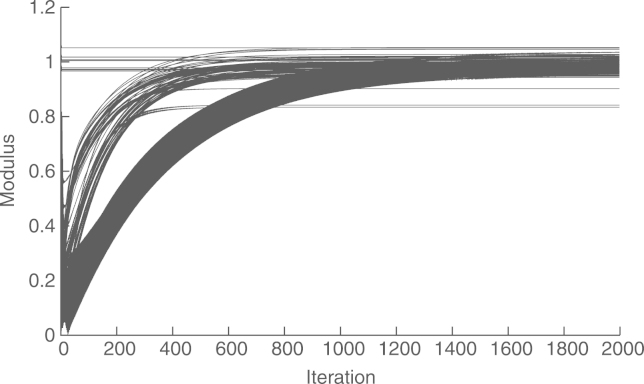
Convergence of the asymptotic method, with preconditioning, in a cyclic network.

**Fig. 5:**
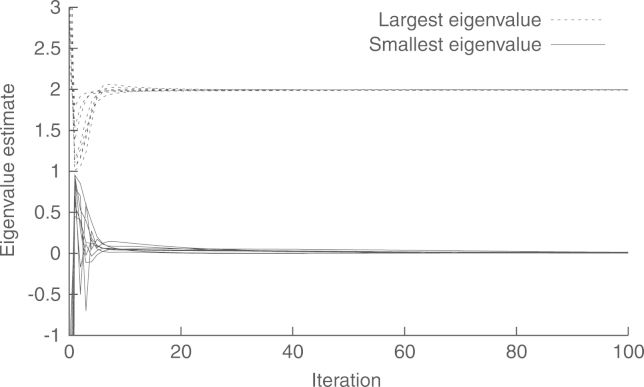
Convergence of the distributed eigenvalue estimation algorithm.

Fig. 3 shows the convergence of the asymptotic method without preconditioning. To this end, we show the modulus of the estimated voltage of each bus at each step. We see that the convergence is very slow, with a relative difference of $r\left(10^{6}\right)=-37\;\mathrm{dB}$, between the global estimate and the one obtained by the distributed algorithm at $t=10^{6}$. The reason for the slow convergence is that the condition number of Ψ is 478 972. The preconditioning matrix in (20) gives a condition number of 700, which leads to a much faster convergence. This is shown in Fig. 4, where $r\left(2\times 10^{3}\right)=-52.47\;\mathrm{dB}$. Fig. 5 shows that the convergence of the estimation of ‖Υ‖ and ${\left\|\Upsilon^{-1}\right\|}^{-1}$, at each node, is much faster than that of the WLS estimation algorithm. Finally, Table 2 shows the complexity at each node. To this end, we measure the number of multiplications in a whole cycle of Algorithms 1–3.

**Table 2:** Complexity at each node, in number of multiplications per iteration, for the cyclic network topology.

Node	1	2	3	4	5	6	7	8

Complexity	5202	8613	10 469	14 848	26 054	11 810	14 352	10 628

**Table 3:** Nodes forming an acyclic network topology.

Node	Buses
1	1, 2, 3, 4, 5, 6, 7, 8, 9, 10, 11, 12, 13, 14, 117
2	23, 25, 26, 27, 28, 29, 31, 32, 113, 114, 115
3	5, 16, 17, 18, 19, 20, 21, 22, 24, 30, 33, 34, 35, 36, 37, 39, 40, 71, 72, 73
4	38, 41, 42, 43, 44, 45, 46, 47, 48, 69, 70, 74, 75, 76, 77, 118
5	49, 50, 51, 54, 65, 66, 68, 78, 79, 80, 81, 82, 95, 96, 97, 98, 99, 116
6	52, 53, 55, 56, 57, 58, 59, 60, 61, 62, 63, 64, 67
7	83, 84, 85, 86, 87, 88, 89, 90, 91, 92, 93, 94, 100, 101, 102, 103, 104, 105, 106, 107, 108, 109, 110, 111, 112

#### 5.1.2 Acyclic network topology

**Fig. 6:**
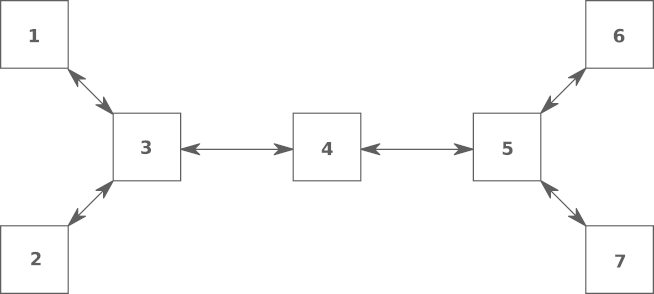
Acyclic network topology induced by the nodes in Table 3.

In the second simulation we do the clustering such that the induced topology is acyclic. From the definition of $B_{i}$, it follows that $j\in B_{i}$ if there is a bus (possibly neither in Node i nor in  j), with a PMU installed, having one neighbor bus (i.e., a bus connected to it via an attached line), including possibly itself, in each node, i and j. The clustering and its induced topology are shown in Table 3 and Fig. 6, respectively.

**Fig. 7:**
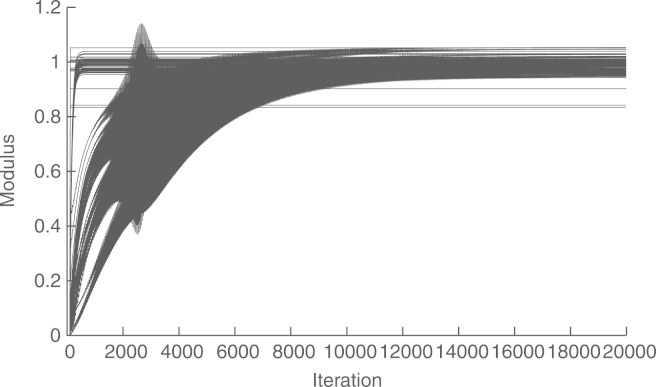
Convergence of the asymptotic method (with preconditioning) in an acyclic network.

**Fig. 8:**
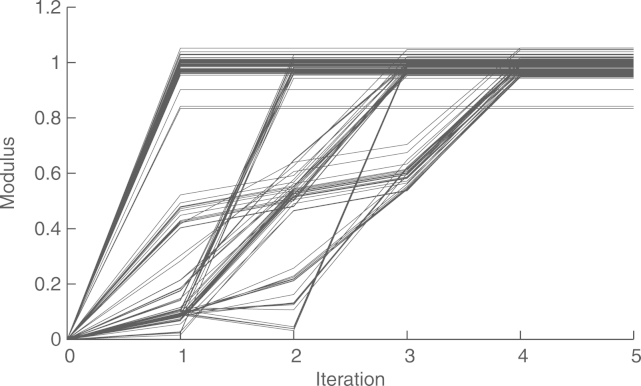
Convergence of the finite-time method (in an acyclic network).

The convergence of the asymptotic method (with preconditioning) is shown in Fig. 7, with a final relative difference with the global estimate of $r\left(20\times 10^{3}\right)=-69.67\;\mathrm{dB}$. In this case, we wait for 100 steps before starting with the distributed estimation. The gap caused by this delay can be seen at the beginning of the graph. We introduced this late start so as to give time for Algorithm 2 and 3 to obtain reasonable approximations of ‖Υ‖ and ${\left\|\Upsilon^{-1}\right\|}^{-1}$, respectively. We see that the asymptotic method presents an oscillating behavior between time steps 1500 and 3500. This is because the transients in the estimation of the scaling factor γ(t) cause the recursions (8) to become temporarily unstable. We also see that the asymptotic method requires about 20×10^3^ steps to converge. This is because in this case, preconditioning leads to a condition number of 5264. On the other hand, the convergence of the finite-time method does not depend on the condition number, but on the network diameter, which in this case is four. Fig. 8 shows the convergence of this method in four steps, with a final error of r(4)=−223.7dB, caused by numerical inaccuracy.

Table 4 shows the complexity at each node. To this end, we consider that solving the positive-definite linear system for computing ${\overset{ˇ}{x}}_{i,j}\left(t\right)$, using the Cholesky decomposition, requires $n^{3}/3+2n^{2}$ multiplications ($n^{3}/3$ for the decomposition and $2n^{2}$ for solving two triangular linear systems). Also, computing the inverse of the matrix ${\overset{ˇ}{\Sigma }}_{i}\left(t\right)$, using also the Cholesky Decomposition, requires $n^{3}/2$ multiplications (Krishnamoorthy & Menon, 2011).

**Table 4:** Complexity at each node, in a number of multiplications per iteration, for the acyclic network topology.

Node	Asymptotic method	Finite-time method
1	4985	1912
2	3645	786
3	14 088	4400
4	10 400	2304
5	17 802	3240
6	3891	1267
7	8919	8437

### 5.2 Sensor localization

Sensor localization refers to the problem of obtaining the locations of each node in a network, based on the knowledge of the locations of a few anchor nodes, a well as the mutual distances between neighbor nodes. A distributed method for carrying out this task is proposed in Khan et al. (2009). This method requires that, for each i=1,…,I, Node i lies inside of at least one triangle defined by three of its neighbors $N_{i}=\left\{j,k,l\right\}$. Then, the coordinates $x_{i}$ of Node i can be written as

(25) $$x_{i}=\underset{j\in N_{i}}{\sum }c_{i,j}x_{i,j}\text{,}$$

where the *barycentric coordinates* $c_{i,j}$ are given by

$$c_{i,j}=\frac{S\left(i\cup N_{i}\setminus j\right)}{S\left(N_{i}\right)}\text{,}$$

with S(i,j,k) denoting the area of the triangle formed by Nodes i, j and k. The latter can be computed using the *Cayley–Menger determinant* as follows

$$S^{2}\left(i,j,k\right)=-\frac{1}{16}\left|\begin{array}{cccc} 0 & 1 & 1 & 1\\ 1 & 0 & d_{i,j}^{2} & d_{i,k}^{2}\\ 1 & d_{i,j}^{2} & 0 & d_{j,k}^{2}\\ 1 & d_{i,k}^{2} & d_{j,k}^{2} & 0 \end{array}\right|\text{,}$$

where $d_{i,j}=\left\|x_{i}-x_{j}\right\|$ denotes the distance between Nodes iand  j.

For each i=1,…,I, we have one equation of the form (25), for each triangle containing Node i. We assume that N such triangles exist for each node. Hence, we have N×I equations. Let $x_{i}\in R^{2}$, i=1,…,I, denote the node coordinates and $a_{j}\in R^{2}$, j=1,…,J, denote those of the anchor nodes. Let also $x^{T}=\left[x_{1}^{T},\ldots ,x_{I}^{T}\right]$ and $a^{T}=\left[a_{1}^{T},\ldots ,a_{J}^{T}\right]$. Then, the aforementioned N×I equations can be written as

$$x=\left(C⊗I_{2}\right)x+\left(D⊗I_{2}\right)a\text{,}$$

or equivalently,

(26) y=Ax,

with $y=\left(D⊗I_{2}\right)a$ and $A=I-C⊗I_{2}$. Due to inaccuracy in distance measurements, (26) can be approximately expressed as in (2). In that case, we can use our proposed distributed method to obtain, at each node, a WLS estimation of its coordinates.

**Fig. 9:**
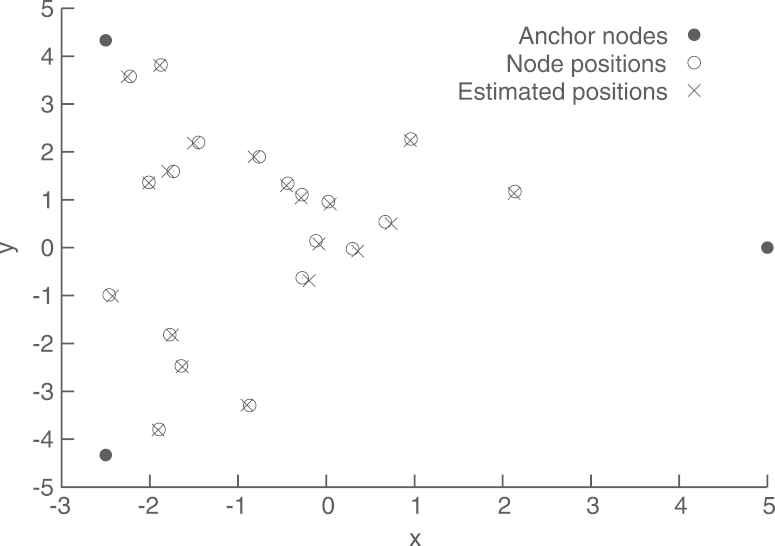
Node positions and estimates.

**Fig. 10:**
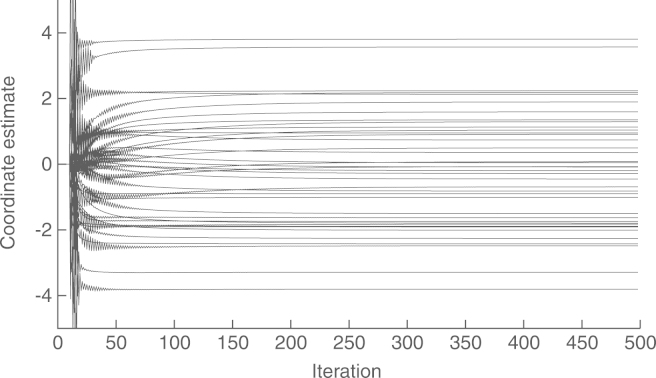
Convergence of the node coordinate estimates.

**Fig. 11:**
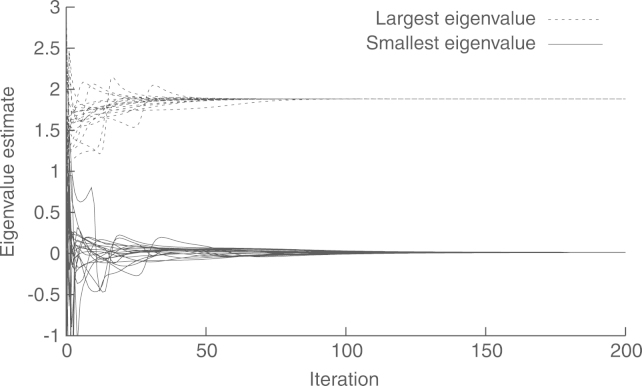
Convergence of the estimated eigenvalues.

The experiment setup is shown in Fig. 9. It includes three anchor nodes, defining a triangle containing I=20 randomly placed nodes. We use a noise covariance matrix $R=\sigma^{2}I_{d}$, where $I_{d}$ denotes the identity matrix, and $\sigma =\sqrt{10^{-3}}≃31.62$ centimeters. With this setup, the global estimate $\overset{ˆ}{x}$ yields a relative localization error of e=−33.39dB, defined as in (23). The convergence of the coordinate estimates at each node, using the proposed method, with preconditioning, is shown in Fig. 10. As before, we wait for 10 steps before starting the iterations, to give time for Algorithms 2 and 3 to obtain reasonable approximations of ‖Υ‖ and ${\left\|\Upsilon^{-1}\right\|}^{-1}$, respectively. The convergences of these estimates are shown in Fig. 11. Finally, the complexity at each node is shown in Table 5.

**Table 5:** Complexity at each sensor node, in a number of multiplications per iteration.

Node	1	2	3	4	5	6	7	8	9	10	11	12	13	14	15	16	17	18	19	20

Complexity	170	282	282	426	170	282	602	602	282	426	282	426	282	170	170	426	426	426	426	426

**Fig. 12:**
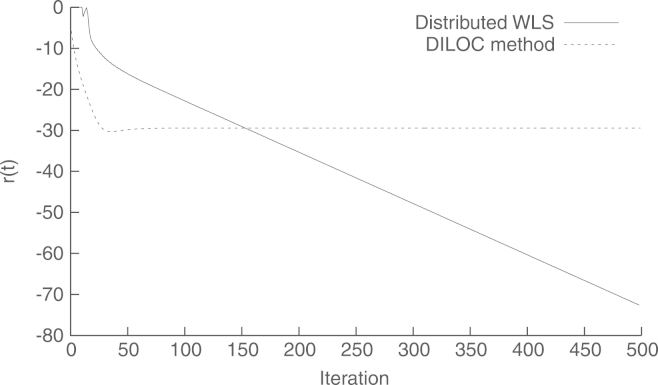
Relative difference with the global estimate $\overset{ˆ}{x}$ vs. iteration t.

For comparison, we also consider the distributed iterative localization algorithm (DILOC) proposed in Khan et al. (2009). This method solves (26) using Richardson’s recursions to invert matrix A. This requires that N=1, i.e., only one equation of the form (25) is considered for each node. In this case, the recursions are guaranteed to converge because, as the authors show, ‖I−A‖<1 holds in this problem. Fig. 12 shows the evolution of the relative difference r(t) (defined as in (24)) between the estimates of each method, and the global estimate $\overset{ˆ}{x}$. We see that the DILOC method has a faster convergence. This is because the condition number of A is smaller than that of $A^{*}R^{-1}A$, which is the matrix inverted by our proposed method. However, at t=500, the DILOC method yields r(500)=−29.44dB, while the proposed one gives r(500)=−72.71dB. This difference results from the fact that the DILOC method does not produce the WLS solution on the limit.^1^

## 6 Conclusion

We proposed two methods for weighted least squares estimation in large-scale systems. Both methods converge to the global solution and aim to maximize the convergence speed. The first method converges asymptotically and involves a distributed estimation of the scaling parameter upon which the convergence speed depends. To further speed up the convergence, we also use a practically feasible preconditioning method, for which we bounded the speed difference with respect to the fastest theoretically achievable. The second proposed method has an even faster convergence, as it achieves the global optimal in finite time. However, it is only suitable for applications where the graph produced by the communication network contains no loops.

## Appendix A Proofs of Section 3

### A.1 Proof of Theorem 6

Fix t∈N. Let $k_{i}\left(t\right)\in R$ and

(A.1) $${\overset{¯}{b}}_{i}\left(t\right)=k_{i}\left(t\right)b_{i}\left(t\right)\text{.}$$

From (14), we have

$${\overset{¯}{b}}_{i}\left(t+1\right)=\frac{k_{i}\left(t+1\right)}{k_{i}\left(t\right)}\Pi_{i}^{1/2}\underset{k\in I_{i}}{\sum }\underset{j\in O_{k}}{\sum }\frac{k_{i}\left(t\right)}{k_{j}\left(t\right)}\overset{\left(k\right)}{\underset{i,j}{\Psi }}\overset{1/2}{\underset{j}{\Pi }}{\overset{¯}{b}}_{j}\left(t\right)\text{.}$$

Let $k_{i}\left(0\right)=1$ and

$$\varsigma_{i}\left(t\right)=\frac{k_{i}\left(t\right)}{k_{i}\left(t-1\right)}\text{,}$$

so that

$$k_{i}\left(t\right)=\overset{t}{\underset{\tau =1}{\prod }}\varsigma_{i}\left(\tau \right)\text{.}$$

Then,

(A.2) $${\overset{¯}{b}}_{i}\left(t+1\right)=\varsigma_{i}\left(t+1\right){\overset{\sim }{b}}_{i}\left(t+1\right)\text{,}$$

with

$${\overset{\sim }{b}}_{i}\left(t+1\right)=\Pi_{i}^{1/2}\underset{k\in I_{i}}{\sum }\underset{j\in O_{k}}{\sum }\upsilon_{i,j}\left(t\right)\overset{\left(k\right)}{\underset{i,j}{\Psi }}\overset{1/2}{\underset{j}{\Pi }}{\overset{¯}{b}}_{j}\left(t\right)\text{,}$$

$$\upsilon_{i,j}\left(t\right)=\overset{t}{\underset{\tau =1}{\prod }}\frac{\varsigma_{i}\left(\tau \right)}{\varsigma_{j}\left(\tau \right)}\text{.}$$

We need to design $k_{i}\left(t+1\right)$, or equivalently $\varsigma_{i}\left(t+1\right)$, to avoid the indefinite increase or decrease of b(t). In principle, this could be achieved by choosing

$$k_{i}\left(t\right)={\left\|b_{i}\left(t\right)\right\|}^{-1}\text{,}$$

so that $\left\|{\overset{¯}{b}}_{i}\left(t\right)\right\|=1$, for all t∈N. From (A.2), this would lead to

$$\varsigma_{i}\left(t+1\right)={\left\|{\overset{\sim }{b}}_{i}\left(t+1\right)\right\|}^{-1}\text{.}$$

However, the question then arises as to whether some of the scalars $\upsilon_{i,j}\left(t\right)$ would grow to infinity. Notice that

$$\upsilon_{i,j}\left(t\right)=\frac{k_{i}\left(t\right)}{k_{j}\left(t\right)}\text{.}$$

Hence, this could only happen if some vector in the eigenspace associated to the largest eigenvalue of Υ has zero components in the entries corresponding to $b_{j}\left(t\right)$. We call a matrix satisfying this property, *ill-posed*. Although the set of ill-posed matrices is nowhere dense, (i.e., it is unlikely to have an ill-posed matrix Υ), we can avoid the indefinite growth of $\upsilon_{i,j}\left(t\right)$ by choosing $\varsigma_{i}\left(t+1\right)$ so that $\left\|{\overset{¯}{b}}_{i}\left(t+1\right)\right\|\leq 1$ and, for all $j\in B_{i}=\left\{j:\Psi_{i,j}\neq 0\right\}$,

$$\varsigma_{i}\left(t+1\right)\upsilon_{i,j}\left(t\right)\leq 1\text{.}$$

This leads to

$$\varsigma_{i}\left(t+1\right)=\min\left\{{\left\|{\overset{\sim }{b}}_{i}\left(t+1\right)\right\|}^{-1},\overset{-1}{\underset{i,j}{\upsilon }}\left(t\right),j\in B_{i}\right\}=\max{\left\{\left\|{\overset{\sim }{b}}_{i}\left(t+1\right)\right\|,\upsilon_{i,j}\left(t\right),j\in B_{i}\right\}}^{-1}\text{.}$$

From (15), (A.1), the estimate ${\overset{¯}{\Upsilon }}_{i}\left(t\right)$ of ‖Υ‖ at t is

$${\overset{¯}{\Upsilon }}_{i}\left(t\right)=\frac{\left\|b_{i}\left(t\right)\right\|}{\left\|b_{i}\left(t-1\right)\right\|}=\varsigma_{i}^{-1}\left(t\right)\frac{\left\|{\overset{¯}{b}}_{i}\left(t\right)\right\|}{\left\|{\overset{¯}{b}}_{i}\left(t-1\right)\right\|}\text{.}$$

However, if Υ is ill-posed, $\left\|{\overset{¯}{b}}_{i}\left(t\right)\right\|$ will tend to zero. In such case, ${\overset{¯}{\Upsilon }}_{i}\left(t\right)$ can be computed by

$${\overset{¯}{\Upsilon }}_{i}\left(t\right)=\varsigma_{j}^{-1}\left(t\right)\frac{\left\|{\overset{¯}{b}}_{j}\left(t\right)\right\|}{\left\|{\overset{¯}{b}}_{j}\left(t-1\right)\right\|}\text{,}$$

for some neighbor node j for which $\left\|{\overset{¯}{b}}_{j}\left(t\right)\right\|$ does not tend to zero. Notice that such a neighbor always exists, for otherwise Node i would be isolated from all other nodes.

### A.2 Proof of Theorem 7

We need use the following result. Lemma 16 *Let*$M={\left[M_{i,j}\right]}_{i,j=1,\ldots ,I}$*be a block symmetric matrix. Then*

$$\left\|M\right\|\leq \underset{i}{\max}\psi_{i}\text{,}$$

*where*

$$\psi_{i}=\overset{I}{\underset{j=1}{\sum }}\left\|M_{i,j}\right\|\text{.}$$

Proof Let y=Mx, with $x=\left[x_{1},\ldots ,x_{I}\right]$ and $y=\left[y_{1},\ldots ,y_{I}\right]$. We have

$$\left\|y_{i}\right\|\leq \overset{I}{\underset{j=1}{\sum }}\left\|M_{i,j}\right\|\left\|x_{j}\right\|=\overset{I}{\underset{j=1}{\sum }}{\left\|M_{i,j}\right\|}^{1/2}{\left(\left\|M_{i,j}\right\|{\left\|x_{j}\right\|}^{2}\right)}^{1/2}\leq {\left(\overset{I}{\underset{j=1}{\sum }}\left\|M_{i,j}\right\|\right)}^{1/2}{\left(\overset{I}{\underset{j=1}{\sum }}\left\|M_{i,j}\right\|{\left\|x_{j}\right\|}^{2}\right)}^{1/2}\text{.}$$

Let $\psi =\max_{i}\psi_{i}$. Then,

$${\left\|y\right\|}^{2}=\overset{I}{\underset{i=1}{\sum }}{\left\|y_{i}\right\|}^{2}\leq \overset{I}{\underset{i=1}{\sum }}\left(\overset{I}{\underset{j=1}{\sum }}\left\|M_{i,j}\right\|\right)\left(\overset{I}{\underset{j=1}{\sum }}\left\|M_{i,j}\right\|{\left\|x_{j}\right\|}^{2}\right)\leq \psi \overset{I}{\underset{i=1}{\sum }}\overset{I}{\underset{j=1}{\sum }}\left\|M_{i,j}\right\|{\left\|x_{j}\right\|}^{2}\leq \psi^{2}\overset{I}{\underset{j=1}{\sum }}{\left\|x_{j}\right\|}^{2}=\psi^{2}{\left\|x\right\|}^{2}\text{,}$$

and the result follows.

Proof of Theorem 7 Using Lemma 16 we have

$$\left\|\Upsilon \right\|\leq \underset{i}{\max}\psi_{i}\text{,}$$

with

$$\psi_{i}=\overset{I}{\underset{j=1}{\sum }}\left\|\Pi_{i}^{1/2}\Psi_{i,j}\Pi_{j}^{1/2}\right\|=\overset{I}{\underset{j=1}{\sum }}\left\|\underset{k:i,j\in O_{k}}{\sum }\overset{1/2}{\underset{i}{\Pi }}\Psi_{i,j}^{\left(k\right)}\Pi_{j}^{1/2}\right\|\leq \overset{I}{\underset{j=1}{\sum }}\underset{k:i,j\in O_{k}}{\sum }\left\|\overset{1/2}{\underset{i}{\Pi }}\Psi_{i,j}^{\left(k\right)}\Pi_{j}^{1/2}\right\|=\underset{k\in I_{i}}{\sum }\underset{j\in O_{k}}{\sum }\left\|\overset{1/2}{\underset{i}{\Pi }}\overset{\left(k\right)}{\underset{i,j}{\Psi }}\overset{1/2}{\underset{j}{\Pi }}\right\|\text{.}$$

Hence,

$$\left\|\Upsilon \right\|\leq \underset{i}{\max}\phi_{i}\text{,}$$

and the result follows.

### A.3 Proof of Theorem 8

We need the following lemma. Lemma 17 *If*$M=\left[\begin{array}{cc} I & C\\ C^{*} & I \end{array}\right]\geq 0$*, then*‖C‖≤1*.*

Proof Let $u^{T}=\left[\begin{array}{cc} x^{T} & y^{T} \end{array}\right]$. Then, for any u,

$$u^{*}Mu={\left\|x\right\|}^{2}+{\left\|y\right\|}^{2}+2y^{T}Cx\geq 0\text{.}$$

Choose x and y such that ‖x‖=‖y‖=1 and $y^{*}Cx=-\left\|C\right\|$. Then, the inequality above becomes 1−‖C‖≥0, and the result follows.

Proof of Theorem 8 Recall that $\Pi =\mathrm{diag}\left\{\Pi_{1},\ldots ,\Pi_{I}\right\}$ and let $D=\overset{\sim }{\Pi }\Pi^{-1}$, with $\overset{\sim }{\Pi }\in P$. Then,

$${\overset{\sim }{\Pi }}^{1/2}\Psi {\overset{\sim }{\Pi }}^{1/2}=D^{1/2}\Upsilon D^{1/2}\text{,}$$

and we have

(A.3) $$\kappa \left({\overset{\sim }{\Pi }}^{1/2}\Psi {\overset{\sim }{\Pi }}^{1/2}\right)=\kappa \left(D^{1/2}\Upsilon D^{1/2}\right)=\kappa^{2}\left({\left(\Upsilon D\right)}^{1/2}\right)\text{.}$$

Let $\bar{\sigma }\left(A\right)$ and $\underline{\sigma }\left(A\right)$ denote the largest and smaller singular values of A, respectively. Now

$$\bar{\sigma }\left({\left(\Upsilon D\right)}^{1/2}\right)=\underset{x\neq 0}{\max}\frac{\left\|{\left(\Upsilon D\right)}^{1/2}x\right\|}{\left\|x\right\|}=\underset{x\neq 0}{\max}\frac{\left\|\Upsilon^{1/2}x\right\|}{\left\|D^{-1/2}x\right\|}\geq \frac{\left\|\Upsilon^{1/2}x_{0}\right\|}{\left\|D^{-1/2}x_{0}\right\|}\text{,}$$

for any $x_{0}\neq 0$. Similarly,

$$\underline{\sigma }\left({\left(\Upsilon D\right)}^{1/2}\right)\leq \frac{\left\|\Upsilon^{1/2}y_{0}\right\|}{\left\|D^{-1/2}y_{0}\right\|}\text{,}$$

for any $y_{0}\neq 0$. Let $x_{0}$ and $y_{0}$ have unit norm and be such that $D^{-1/2}x_{0}=\underline{\sigma }\left(D^{-1/2}\right)x_{0}$ and $\Upsilon^{1/2}y_{0}=\underline{\sigma }\left(\Upsilon^{1/2}\right)y_{0}$. Then,

$$\kappa \left({\left(\Upsilon D\right)}^{1/2}\right)=\frac{\bar{\sigma }\left({\left(\Upsilon D\right)}^{1/2}\right)}{\underline{\sigma }\left({\left(\Upsilon D\right)}^{1/2}\right)}\geq \frac{\left\|D^{-1/2}y_{0}\right\|}{\underline{\sigma }\left(D^{-1/2}\right)}\frac{\left\|\Upsilon^{1/2}x_{0}\right\|}{\underline{\sigma }\left(\Upsilon^{1/2}\right)}\geq \frac{\left\|\Upsilon^{1/2}x_{0}\right\|}{\underline{\sigma }\left(\Upsilon^{1/2}\right)}\text{.}$$

Now, since $D^{-1/2}$ is block diagonal, $x_{0}$ can be chosen so that its nonzero components correspond to only one block of $D^{-1/2}$. Let $x_{0,b}$ denote the entries of $x_{0}$ in that block, and $\Upsilon_{b}^{1/2}$ denote the columns of $\Upsilon^{1/2}$ corresponding to the same block. Then,

$${\left\|\Upsilon^{1/2}x_{0}\right\|}^{2}=\left|{\left(\Upsilon^{1/2}x_{0}\right)}^{*}\Upsilon^{1/2}x_{0}\right|=\left|x_{0,b}^{*}\Upsilon_{b}^{1/2}\Upsilon_{b}^{1/2}x_{0,b}\right|=\left|x_{0,b}^{*}x_{0,b}\right|={\left\|x_{0}\right\|}^{2}=1\text{.}$$

Then,

$$\kappa \left({\left(\Upsilon D\right)}^{1/2}\right)\geq \frac{1}{\underline{\sigma }\left(\Upsilon^{1/2}\right)}=\frac{\kappa \left(\Upsilon^{1/2}\right)}{\bar{\sigma }\left(\Upsilon^{1/2}\right)}\text{.}$$

Let $\Upsilon ={\left[\Upsilon_{i,j}\right]}_{i,j=1,\ldots ,I}$ be the block partition of Υ. From Lemma 17, $\left\|\Upsilon_{i,j}\right\|\leq 1$, for all i,j=1,…,I. Then, from Lemma 16, Lemma 17

$$\bar{\sigma }\left(\Upsilon \right)\leq \underset{i}{\max}\overset{I}{\underset{j=1}{\sum }}\left\|\Upsilon_{i,j}\right\|\leq \underset{i}{\max}\left|B_{i}\right|=\beta \text{.}$$

Hence, from (A.3),

$$\kappa \left({\overset{\sim }{\Pi }}^{1/2}\Psi {\overset{\sim }{\Pi }}^{1/2}\right)\geq \frac{\kappa \left(\Upsilon \right)}{\bar{\sigma }\left(\Upsilon \right)}\geq \frac{\kappa \left(\Upsilon \right)}{\beta }\text{.}$$

The result follows since the inequality above holds for any $\overset{\sim }{\Pi }$.

## Appendix B Proofs of Section 4

Proof of Lemma 12 From Kailath, Sayed, and Hassibi (2000, A.1(v)), we have $\Psi_{a,a}-\Psi_{a,b}{\overset{ˇ}{\Sigma }}_{b}\Psi_{b,a}$ and $\Psi_{b,b}-\Psi_{b,a}\Psi_{a,a}\Psi_{a,b}$ are invertible, and

$$\Psi^{-1}=\left[\begin{array}{cc} \Sigma_{a} & -\Sigma_{a}\Psi_{a,b}\Psi_{b,b}^{-1}\\ -\Sigma_{b}\Psi_{b,a}\Psi_{a,a}^{-1} & \Sigma_{b} \end{array}\right]$$

with $\Sigma_{a}={\left(\Psi_{a,a}-\Psi_{a,b}\Psi_{b,b}^{-1}\Psi_{b,a}\right)}^{-1}$ and $\Sigma_{b}={\left(\Psi_{b,b}-\Psi_{b,a}\Psi_{a,a}^{-1}\Psi_{a,b}\right)}^{-1}$. The result then follows from (3).

Proof of Lemma 13 Follows immediately by applying Lemma 12 with $x_{a}=x_{1}$ and $x_{b}^{T}=\left[x_{2},\ldots ,x_{I}\right]$. Before proving Theorem 15, we introduce some notation. Notation 18 *For each*i∈I={1,…,I}*, and*$j\in B_{i}$*, let*$M_{i}\left(0\right)=M_{j,i}\left(0\right)=\left\{i\right\}$*. Then, for each*t∈N*, define recursively the following two sequences of sets*

$$M_{i}\left(t\right)=\underset{k\in M_{i}\left(t-1\right)}{⋃}B_{k}\text{,}$$

$$M_{j,i}\left(t\right)=\underset{k\in M_{j,i}\left(t-1\right)}{⋃}B_{k}\setminus \left\{j\right\}\text{.}$$

*I.e.,*$M_{i}\left(t\right)$*is the set of indexes of nodes which are*t*edges away from Node*i*, and*$M_{j,i}\left(t\right)$*is the set resulting after removing from*$M_{i}\left(t\right)$*the indexes of those nodes which are linked to Node*i*through Node*j*. For each*$t\in N_{0}=N\cup \left\{0\right\}$*and*i∈I*, let*$\xi_{i}^{T}\left(t\right)=\left[x_{k}^{T}:k\in M_{i}\left(t\right)\right]$*,*$\xi_{i,j}^{T}\left(t\right)=\left[x_{k}^{T}:k\in M_{i,j}\left(t\right)\right]$*,*$\Omega_{i}\left(t\right)={\left[A_{k,l}\right]}_{k\in I,l\in M_{i}\left(t\right)}$*and*$\Omega_{i,j}\left(t\right)={\left[A_{k,l}\right]}_{k\in I,l\in M_{i,j}\left(t\right)}$*. Also, let*$\left({\overset{ˆ}{\xi }}_{i}\left(t\right),\Xi_{i}\left(t\right)\right)$*be the WLS solution of the reduced system*

(B.1) $$y=\Omega_{i}\left(t\right)\xi_{i}\left(t\right)+v\text{,}$$

*i.e.,*

$${\overset{ˆ}{\xi }}_{i}\left(t\right)=\Xi_{i}\left(t\right)\Omega_{i}^{*}\left(t\right)R^{-1}y$$

$$\Xi_{i}\left(t\right)={\left(\Omega_{i}^{*}\left(t\right)R^{-1}\Omega_{i}\left(t\right)\right)}^{-1}$$

*and*$\left({\overset{ˆ}{\xi }}_{i,j}\left(t\right),\Xi_{i,j}\left(t\right)\right)$*be WLS the solution of*

$$y=\Omega_{i,j}\left(t\right)\xi_{i,j}\left(t\right)+v\text{.}$$

Proof of Theorem 15 Suppose that, at time t∈N, and for each i∈I and $j\in B_{i}\setminus \left\{i\right\}$, Node i is able to compute the components $\left({\overset{ˇ}{x}}_{i}\left(t\right),{\overset{ˇ}{\Sigma }}_{i}\left(t\right)\right)$, corresponding to the state $x_{i}$, of the solution $\left({\overset{ˆ}{\xi }}_{i}\left(t\right),{\overset{ˆ}{\Xi }}_{j}\left(t\right)\right)$, and the components $\left({\overset{ˇ}{x}}_{i,j}\left(t\right),{\overset{ˇ}{\Sigma }}_{i,j}\left(t\right)\right)$, corresponding to the same state, of $\left({\overset{ˇ}{\xi }}_{i,j}\left(t\right),{\overset{ˇ}{\Xi }}_{i,j}\left(t\right)\right)$. Since the network is acyclic, for each i∈I and each t∈N, we have

$$M_{i}\left(t+1\right)=\left\{i\right\}\cup \underset{j\in B_{i}\setminus \left\{i\right\}}{⋃}M_{i,j}\left(t\right)\text{.}$$

Then, given that Node i receives $\gamma_{i,j}\left(t\right)$ and $\Gamma_{i,j}\left(t\right)$, from each $j\in B_{i}\setminus \left\{i\right\}$, $\left|B_{i}\right|-1$ applications of Lemma 13 (|S| denotes the number of elements in the set S), gives that $\Psi_{i,i}-\sum_{j\in B_{i}\setminus \left\{i\right\}}\Gamma_{j,i}\left(t-1\right)$ is invertible, and Node i is able to compute $\left({\overset{ˇ}{x}}_{i}\left(t+1\right),{\overset{ˇ}{\Sigma }}_{i}\left(t+1\right)\right)$. Also, $\left|B_{i}\right|-2$ applications of Lemma 13 give that, for each $j\in B_{i}\setminus \left\{i\right\}$, $\Psi_{i,i}-\sum_{j\in B_{i}\setminus \left\{i,j\right\}}\Gamma_{j,i}\left(t-1\right)$ is invertible and Node i can compute $\left({\overset{ˇ}{x}}_{i,j}\left(t+1\right),{\overset{ˇ}{\Sigma }}_{i,j}\left(t+1\right)\right)$. Then, the result follows after initializing the induction above using $\left({\overset{ˇ}{x}}_{i}\left(0\right),{\overset{ˇ}{\Sigma }}_{i}\left(0\right)\right)$, at each i∈I, for which no information exchange is required. At each $t\in N_{0}$ and i∈I, $\left({\overset{ˇ}{x}}_{i}\left(t\right),{\overset{ˇ}{\Sigma }}_{i}\left(t\right)\right)$ is the WLS solution of the sub-system (B.1). Since (B.1) is obtained by considering only the nodes in $M_{i}\left(t\right)$, and $M_{i}\left(t\right)=I$, for all $t\geq \rho_{i}$, (22) follows.

## Footnotes

1 Notice that, in the scenario considered in this work, noisy inter-node distances are only measured once, and they remain unchanged during the whole iteration process. This is in contrast to the scenario considered in Khan et al. (2009), where these distances are re-measured at each iteration.
